# Setting Teeth in Artificial Sockets

**Published:** 1886-09

**Authors:** S. S. Southworth

**Affiliations:** Sacramento, Cal.


### SETTING TEETH IN ARTIFICIAL SOCKETS.

BY DR. S. S. SOUTHWORTH, SACRAMENTO, CAL.

I have recently spent a few hours in Dr. Younger's company, talking over his numerous operations, his methods, failures and successes. I have talked with other dentists in San Francisco who have seen his operations in transplantation in natural and artificial sockets, and they all pronounce them successful. One of the dentists had worn one of the replantations for a year, and it was apparently as firm as the contiguous ones. In this case, if I remember correctly, a root was extracted, the canal filled, an artificial crown put on, and it was then replaced in the socket. This may not be a new procedure, but at least it is an ingenious operation, and one that is properly appreciated by the patient.

Learning that Dr. Younger would give a clinic at the State Society meeting, my son went to San Francisco last week, and was in attendance during the session. He returned very much interested in the "gimlet hole" business, and already has a patient prepared to sacrifice the inestimable comforts of a lateral incisor on a rubber plate. I take down his own words as a witness of Dr. Younger's method:

"I was present at Dr. Younger's clinic on Wednesday of last week, and was greatly surprised at the simplicity of the operation. The patient was a San Francisco dentist, who lost a left superior first bicuspid, fourteen years ago. The tooth used for implanting had, three weeks before, been extracted from the over-crowded arch of a lady patient. The pulp cavity and canal was filled with Hill's stopping, and the hole in the crown with gold. The apical end of the canals were closed with gold screws, the ends being smoothed and neatly polished. It was kept in one of William's cylinder bottles filled with a liquid composed of two minims of merc. chloride and one thousand of water. The bottle rested in a pan of warm water kept at a temperature of 110° to 115°.

"The first operation was gouging out a piece of the gum with sharp chisels, corresponding to the size of the required socket, but a trifle less in diameter. After this the gum was dissected from the alveolus for a few lines in all directions. A trepan of suitable size was then placed in the engine and a socket cut about half the length of the tooth. The cutting instrument had a diameter equal to the antero-posterior diameter of the root, and two adjoining cuts were made, one on the lingual and one on the labial side of the socket, having no partition between them, and leaving a socket approximating the shape of the tooth. The debris was syringed out with a cold solution of the aqua-mercuric liquid from a large bottle standing on a side table. The upper part of the socket was cut out with a smaller trephine and shaped to receive the end of the bifurcated root. The after shaping was done with conical shaped corundum points.

"The space between the adjoining cuspid and bicuspid being narrow, the crown of the borrowed tooth was ground off so as to slip into its new position without crowding. Frequent applications were made to see if the tooth fitted snugly, and the tooth always replaced in the warm solution. At the end of an hour and a half the doctor dried out the socket, placed the tooth in position and pushed it home with a crotched stick, as one would do with a pivot operation, and invited the forty dentists present to inspect it. In my examination I found the tooth so solid that it was impossible to remove it without an instrument. It was whiter than the adjoining teeth, but time will change the color.

"I saw Dr. Cummings, the dentist operated on, the following day, and every day during the week, and he assured me he had suffered more pain in getting a tooth filled than the whole operation caused, and was agreeably surprised at the small amount of inflammation which had followed this novel case of heroic surgery.

"Recognizing that the success of an operation depends on the results obtained, Dr. Younger had a patient at hand for whom a similar operation had been performed over a year ago. The patient was a married lady, I should think thirty-five years of age. In this case a superior second bicuspid had been implanted in an artificial socket, and the owner of it was so proud of the success that she took very great pleasure in not only allowing us to look at it and test its stability, but also in exhibiting two inferior molars which she was carrying around in her pocket-book, subjects for Dr. Younger's manipulative skill in the near future. The color of the bicuspid was perfect, and I could not distinguish any difference between it and its neighbors.

"I am too young a practitioner to discuss the question as to whether or not bone cells can be produced without the aid of periosteum, but I am satisfied that teeth can be implanted in "gimlet holes" in the alveolus and become solid in their attachment, and I further believe that a bone formation does take place around the root, filling the interstitial spaces.

"I learn on the most credible authority that Dr. Younger has nearly one hundred successful cases of this character, and I know that he is giving this practice his principal attention. If you visit his office now, he will show you a box of teeth which he has collected from his professional friends and during his own practice, and he is ready to select one for any case which presents itself, without regard to how long the tooth to be implanted has been extracted, so long as the dried pericementum is attached."

I write this in a hurry, for I think you ought to have it, and you can depend upon it as being in accordance with facts.

